# Structural insights into the triple agonism at GLP-1R, GIPR and GCGR manifested by retatrutide

**DOI:** 10.1038/s41421-024-00700-0

**Published:** 2024-07-17

**Authors:** Wenzhuo Li, Qingtong Zhou, Zhaotong Cong, Qingning Yuan, Wenxin Li, Fenghui Zhao, H. Eric Xu, Li-Hua Zhao, Dehua Yang, Ming-Wei Wang

**Affiliations:** 1 State Key Laboratory of Chemical Biology, Shanghai Institute of Materia Medica, Chinese Academy of Sciences, Shanghai, China; 2 The National Center for Drug Screening, Shanghai Institute of Materia Medica, Chinese Academy of Sciences, Shanghai, China; 3 University of Chinese Academy of Sciences, Beijing, China; 4 Department of Pharmacology, School of Basic Medical Sciences, Fudan University, Shanghai, China; 5 State Key Laboratory of Drug Research, Center for Structure and Function of Drug Targets, Shanghai Institute of Materia Medica, Chinese Academy of Sciences, Shanghai, China; 6 Research Center for Deepsea Bioresources, Sanya, Hainan China; 7 Department of Chemistry, School of Science, The University of Tokyo, Tokyo, Japan; 8 Engineering Research Center of Tropical Medicine Innovation and Transformation of Ministry of Education, School of Pharmacy, Hainan Medical University, Haikou, Hainan China

**Keywords:** Cryoelectron microscopy, Hormone receptors

Dear Editor,

The global prevalence of type 2 diabetes and obesity, affecting over 507 million^1^ and 890 million^2^ individuals, respectively, underscores the urgent need for more effective treatments. The most successful treatments currently available include glucagon-like peptide-1 (GLP-1) receptor agonists (GLP-1RAs), exemplified by semaglutide (approved in 2017, $19.9 billion sales in 2023)^3^ and tirzepatide (approved in 2022, $5.3 billion sales in 2023)^4^. Unlike semaglutide which solely activates GLP-1R (one of the three pivotal receptors regulating glucose homeostasis), tirzepatide also activates glucose-dependent insulinotropic polypeptide (GIP) receptor (GIPR) to enhance metabolic benefits with reduced side-effects^5^. However, both medications do not target glucagon (GCG) receptor (GCGR). Recently, retatrutide (also known as LY3437943)^6–9^ has shown impressive efficacy in obesity treatment through triple agonism at GLP-1R, GIPR and GCGR. Compared to the corresponding endogenous hormones, retatrutide is more potent at GIPR by a factor of 8.9, and less potent at GCGR and GLP-1R by factors of 0.3 and 0.4, respectively^7^ (Fig. 1a). Retatrutide induces greater body weight losses in obese mice than tirzepatide, due to an increased energy expenditure through GCGR activation^6^. In a phase 2 obesity trial, retatrutide demonstrated an average weight loss of 17.5% at 24 weeks and 24.2% at 48 weeks in the 12 mg dose group^7^. Additionally, in a phase 2 trial targeting type 2 diabetes, retatrutide demonstrated significant improvements in glycemic control and substantial weight reduction, maintaining a safety profile comparable to certain approved GLP-1RAs^8^ (Supplementary Table S1). These results support the rationale of developing retatrutide as an alternative to semaglutide and/or tirzepatide^5^. Indeed, phase 3 clinical trials of retatrutide for type 2 diabetes, non-alcoholic fatty liver disease, and obesity are presently underway (Supplementary Table S2)^10^. To understand the multiplexed pharmacological actions of retatrutide, we used cryo-electron microscopy (cryo-EM) to determine the structures of GLP-1R, GIPR and GCGR bound to retatrutide.

**Fig. 1:**
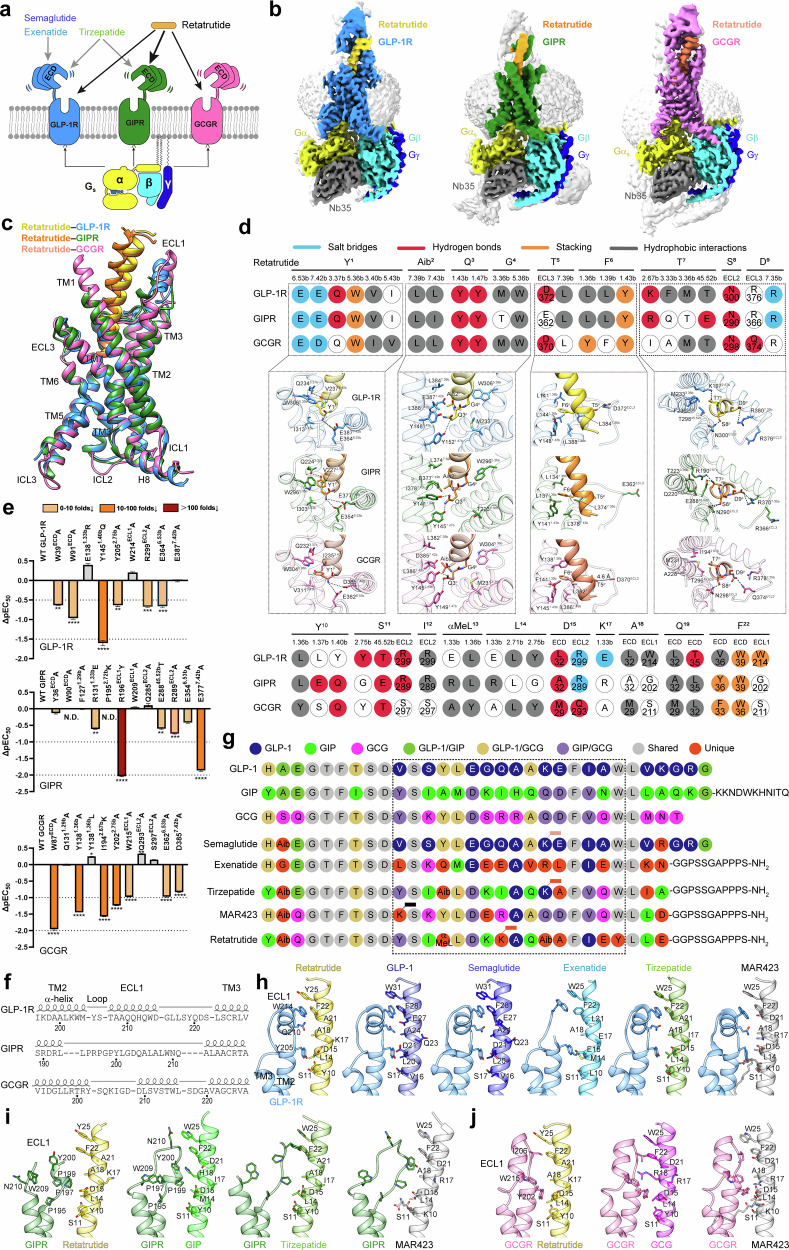
Molecular recognition of retatrutide by GLP-1R, GIPR and GCGR. **a** Retatrutide (LY3437943) possesses combinatorial agonism at GLP-1R, GIPR and GCGR, distinct from the approved GLP-1R agonists (such as semaglutide and exenatide) and GLP-1R/GIPR dual agonist tirzepatide. **b** Cryo-EM maps of retatrutide-bound GLP-1R (left), GIPR (middle) and GCGR (right) in complex with G_s_. The colored cryo-EM density maps are shown at the thresholds of 0.110, 0.107 and 0.134 for the retatrutide–GLP-1R–G_s_, retatrutide–GIPR–G_s_ and retatrutide–GCGR–G_s_ complexes, respectively. The GLP-1R is shown in dodger blue, GIPR in forest green, GCGR in hot pink, GLP-1R-bound retatrutide in gold, GIPR-bound retatrutide in orange, GCGR-bound retatrutide in coral, Gα_s_ in yellow, Gβ subunit in cyan, Gγ subunit in blue and Nb35 in gray. **c** Structural comparison of retatrutide–GLP-1R, retatrutide–GIPR and retatrutide–GCGR. Receptor ECD and G protein are omitted for clarity. **d** Schematic diagram of retatrutide recognition mode in GLP-1R, GIPR and GCGR, described by fingerprint strings encoding different interaction types of the surrounding residues in each receptor. Close-up views of the interactions are shown for the N-terminal nine residues of retatrutide. The polar contacts are shown as black dashed lines. Receptor residues are labeled with class B1 GPCR numbering and colored sky blue for salt bridge, red for hydrogen bond, orange for stacking and gray for hydrophobic interactions. Residues that show no interaction with retatrutide are displayed as white circles. **e** Effects of GLP-1R (top), GIPR (middle), and GCGR (bottom) mutations on retatrutide-induced cAMP accumulation. Bars represent differences in the calculated retatrutide potency (pEC_50_) for representative mutants relative to the wild-type (WT). Data are colored according to the extent of effect. Data shown are from at least three independent experiments performed in quadruplicate. All data were analyzed by one-way ANOVA and Dunnett’s test. *P < 0.05, **P < 0.01, ***P < 0.001, and ****P < 0.0001. N.D., values that could not be determined due to incomplete curve fits. **f** The distinct secondary structures of the ECL1 of GLP-1R, GIPR and GCGR. **g** Amino acid sequence comparison of endogenous hormones, representative dual and triple agonists. Residues are colored according to sequence conservation among GLP-1, GIP and GCG. Aib α-amino isobutyric acid, αMeL α-methyl L-leucine. Semaglutide, tirzepatide, peptide 20 (MAR423) and retatrutide are acylated with various fatty diacid moieties via a linker connected to the lysine residues at the positions of 20, 20, 10 and 17, respectively. **h** Comparison of the interactions between GLP-1R ECL1 and representative peptide agonists including retatrutide, GLP-1 (PDB ID: 6X18), semaglutide (PDB ID: 7KI0), exenatide (PDB ID: 7LLL), tirzepatide (PDB ID: 7FIM) and peptide 20 (PDB ID: 7VBH). **i** Comparison of the interactions between GIPR ECL1 and representative peptide agonists including retatrutide, GIP (PDB ID: 7DTY), tirzepatide (PDB ID: 7FIY) and peptide 20 (PDB ID: 7FIN). **j** Comparison of the interactions between GCGR ECL1 and representative peptide agonists including retatrutide, GCG (PDB ID: 6LMK) and peptide 20 (PDB ID: 7V35).

Following our previously reported protocols with the NanoBiT tethering strategy^11^, the structures of retatrutide–GLP-1R–G_s_, retatrutide–GIPR–G_s_ and retatrutide–GCGR–G_s_ were determined using the single-particle cryo-EM with overall resolutions of 2.68 Å, 3.26 Å and 2.84 Å, respectively (Fig. 1b; Supplementary Figs. S1, S2 and Table S3). Apart from the α-helical domain of Gα_s_ and the extracellular domain (ECD) of GIPR, the presence of retatrutide, each individual receptor and heterotrimeric G_s_ in the respective complexes were clearly visible in all three cryo-EM maps, thereby allowing unambiguous modeling of the secondary structure and side-chain orientation of all major components (Supplementary Fig. S3). Several segments in the ECD of GCGR, the stalk between ECD and transmembrane helix 1 (TM1) of GLP-1R (residues 130–135), extracellular loop 1 (ECL1) of GIPR (residues 202–207), and intracellular loop 3 (ICL3) of GLP-1R and GIPR (residues 338–343 and 330–333, respectively) were poorly resolved and not modeled in the structures.

Retatrutide adopts a single continuous helix that penetrates the core of the receptor transmembrane domain (TMD) via its N-terminal segment (residues 1–13), while its C-terminal segment (residues 14–30) interacts with the N-terminal α-helix of the ECD, the extracellular tip of TM1 and ECL1 (Fig. 1b–e). Although the overall structures of retatrutide-bound GLP-1R, GIPR and GCGR are highly similar, with Cα root mean square deviation (RMSD) values of 0.88–0.93 Å, unique structural features were noted at ECL1, ECL3 and the extracellular tips of TM1, TM3 and TM7, displaying receptor-specific positions and conformations. Specifically, the extracellular half of TM7 in GCGR shifts outward by 4.56 Å and 3.02 Å (measured by the Cα of R^7.35b^, class B1 GPCR numbering in superscript^12^), facilitating the outward orientation of retatrutide’s N-terminus by 2.84 Å and 2.61 Å (measured by the Cα of Y1^P^, P in superscript indicates peptide residue) compared to these residues at retatrutide–GLP-1R and retatrutide–GIPR structures, respectively. In GLP-1R and GCGR, ECL1 forms a short α-helix structure adjacent to the extracellular tips of TM2 in GLP-1R and TM3 in GCGR (Fig. 1f). Conversely, the ECL1 of GIPR adopts an unwound and relaxed loop conformation, likely due to the presence of three proline residues (P195, P197 and P199). This conformation causes retatrutide to straighten, shifting its tip towards the TMD core by 4.29 Å and 4.13 Å (measured by the Cα of L27^P^), relative to GLP-1R and GCGR, respectively.

Analyzing molecular recognition of retatrutide by GLP-1R, GIPR and GCGR highlights that the triple agonism is achieved through a combination of maintaining common interactions with conserved residues and accommodating receptor-specific residues with variable contacts primarily in the upper half of the TMD pocket (Fig. 1d; Supplementary Fig. S4 and Table S4). These common interactions involve two salt bridges with E^6.53b^ and E/D^7.42b^ (via the positively charged N-terminal nitrogen atom of Y1^P^), stacking interactions with W39^GLP-1R^/W39^GIPR^/W36^GCGR^ (via F22^P^), Y^1.43b^ (via F6^P^) and W^5.36b^ (via Y1^P^), multiple hydrogen bonds with L32^GLP-1R^/A32^GIPR^/M29^GCGR^ (via D15^P^), Y^1.43b^ (via Q3^P^), Y^1.47b^ (via Q3^P^), T/E^45.52b^ (via S11^P^) and N300^GLP-1R^/N290^GIPR^/N298^GCGR^ (via S8^P^), along with extensive hydrophobic contacts with L/Y^1.36b^ (via Y10^P^ and αMeL13^P^), I/V^3.40b^ (via Y1^P^), W^5.36b^ (via G4^P^), L^7.39b^ and I/L^7.43b^ (via Aib2^P^). Consistently, alanine mutations at E^6.53b^ decreased retatrutide-induced cAMP signaling potency by 3.6-fold for GLP-1R, 1.6-fold for GIPR and 8.3-fold for GCGR, while at E/D^7.42b^, potency decreased by 71.6-fold for GIPR and 5.8-fold for GCGR (Fig. 1e; Supplementary Fig. S5 and Table S5). Regarding receptor-specific interactions, the retatrutide–GLP-1R complex displays three salt bridges (D9^P^−R^7.35b^, D15^P^–R299^ECL2^ and K17^P^–E^1.33b^), with the first two also present in the retatrutide–GIPR complex, while the last one is absent in GIPR due to the positively charged R131^1.33b^. Additionally, in GLP-1R and GIPR, K/R^2.67b^ and R299^GLP-1R^/R289^GIPR^ in ECL2 form two hydrogen bonds with T7^P^ and S11^P^, respectively. Conversely, equivalent residues in GCGR (I194^2.67b^ and S297^ECL2^) are unable to form these hydrogen bonds. These observations were supported by our mutagenesis studies, where mutations R299^ECL2^A in GLP-1R, R289^ECL2^A in GIPR and I194^2.67b^K in GCGR caused variable decreases in retatrutide potency (by 3.7-fold, 4.6-fold and 36.0-fold, respectively), while S297^ECL2^A in GCGR slightly increased retatrutide potency. Of note, E138^1.33b^R in GLP-1R and R131^1.33b^E in GIPR modestly increased and decreased the potency of retatrutide, respectively, indicating the receptor-specific adaptability of peptide recognition at this site (Fig. 1e). To compensate, retatrutide establishes GCGR-specific interactions including stacking with F33^ECD^ (via F22^P^) and Y138^1.36b^ (via F6^P^), and three hydrogen bonds with Q142^1.40b^ (via Y10^P^), Q293^ECL2^ (via D15^P^) and Q374^ECL3^ (via D9^P^). The Y138^1.36b^A mutation in GCGR greatly decreased the potency of retatrutide-induced cAMP signaling by 26.9-fold, while Y138^1.36b^L moderately increased it, suggesting the hydrophobic interactions at 1.36b play a crucial role (Fig. 1e). In GIPR, Q138^1.40b^ and two glutamic acid residues (E135^1.37b^ and E288^45.52b^) make three hydrogen bonds with Y10^P^ and T7^P^, while F22^P^ and αMeL13^P^/L14^P^ in retatrutide interact with Y36^ECD^ and R131^1.33b^ through stacking and hydrophobic contacts, respectively. Consistently, the E288^45.52b^T mutation in GIPR reduced retatrutide-induced cAMP accumulation by 3.0-fold. Moreover, R196^ECL1^Y diminished retatrutide potency by 107.7-fold, whereas P195^2.72b^K markedly decreased GIPR-mediated cAMP accumulation, supporting a key role of GIPR ECL1 in retatrutide agonism (Fig. 1e; Supplementary Fig. S5 and Table S5).

Sequence comparison among endogenous hormones, approved drugs and triple agonists revealed that the peptide sequence is conserved in the N-terminal region (residues 4–9), with variations primarily occurring in the middle region (residues 10–21) (Fig. 1g). This middle region is specifically recognized by ECL1, ECL2, and the extracellular tip of TM1, all of which exhibit receptor-specific conformation and structural flexibility (Fig. 1g–j). For instance, ECL1 that primarily interacts with six peptide positions (11^P^, 14^P^, 15^P^, 18^P^, 22^P^ and 25^P^) maintains a robust short α-helix adjacent to TM2 for GLP-1R and TM3 for GCGR, respectively, regardless of the bound peptides (Fig. 1h–j). Therefore, the interacting peptide must possess complementary amino acids at specific positions to align with the rigid ECL1 structure to effectively bind and activate GLP-1R or GCGR. In contrast, the ECL1 of GIPR displays notable flexibility, accommodating a variety of conformations to recognize peptides and allowing for sequence variability. This requirement of ECL1 recognition drives the above six key positions in dual or triple agonists (tirzepatide, peptide 20 (MAR423), and retatrutide) to use amino acids similar to those in GLP-1/GCG, while the other positions in the middle region show considerable variability (Fig. 1g). Of note, leveraging the two conserved segments (residues 4–9, 11, 14, 15, 18, 22 and 25), retatrutide and peptide 20 present a distinct design strategy, developed from the GIP and GCG backbones, respectively, to allow simultaneous agonism at the three pivotal receptors^1,13^. These findings underscore the potential for peptide sequence optimization to fine-tune receptor selectivity or balanced activation, providing tailored approaches for treating different diseases^14^.

In conclusion, our study provides valuable structural insights into the mechanism underlying the superior clinical efficacy of retatrutide, a unimolecular triple agonist simultaneously activating GLP-1R, GIPR and GCGR. The cryo-EM structures of retatrutide-bound GLP-1R, GIPR and GCGR reveal a combination of conserved peptide–receptor interactions and receptor-specific conformations, particularly in ECL1, that enables retatrutide to execute agonism at multiple receptors. Comparison with the binding modes of other dual and triple agonists highlight that the N-terminal and C-terminal regions of the peptide confer receptor selectivity, whereas the middle region offers opportunities for sequence optimization to refine receptor engagement, thereby presenting a useful template for designing better unimolecular therapeutics.

### Supplementary information

Supplementary Information

## Data Availability

All relevant data are available from the authors and/or included in the manuscript or Supplementary Information. The atomic coordinates have been deposited in the Protein Data Bank (PDB) under accession codes: 8YW3 (retatrutide−GLP-1R−G_s_ complex), 8YW4 (retatrutide−GIPR−G_s_ complex) and 8YW5 (retatrutide−GCGR−G_s_ complex); and the electron microscopy maps have been deposited in the Electron Microscopy Data Bank (EMDB) under accession codes: EMD-39621 (retatrutide−GLP-1R−G_s_ complex), EMD-39622 (retatrutide−GIPR−G_s_ complex) and EMD-39623 (retatrutide−GCGR−G_s_ complex).
