# Supplementary material for: Structural insights into the triple agonism at GLP-1R, GIPR and GCGR manifested by retatrutide
Source: Cell Discov. 2024 Jul 17;10:77. doi: 10.1038/s41421-024-00700-0 (PMC11255275; doi:10.1038/s41421-024-00700-0)
Supplement: Supplementary file 1 — Supplementary Information [file 41421_2024_700_MOESM1_ESM.pdf]

Supplementary Information

Materials and Methods

Cell culture

Spodoptera frugiperda (*Sf*9) and HighFive insect cells (Expression Systems) were grown in ESF 921 serum-free medium (Expression Systems) at 27°C and 120 rpm.

Construct

The human GLP-1R and GCGR were cloned into pFastBac vector (Invitrogen) and modified with their native signal sequences replaced by the hemagglutinin (HA) signal peptide to facilitate receptor expression. The NanoBiT tethering strategy was used for GLP-1R–G_s complex[1], in which the C terminus of GLP-1R was directly attached to LgBiT subunit followed by a tobacco etch virus (TEV) protease cleavage site and a double MBP tag. An engineered G_s construct (G112)[2] was used to help stabilize the retatrutide–GLP-1R–G_s complex. Rat Gβ1 was cloned with a C-terminal HiBiT (Promega) connected with a 15-amino acid (15AA) polypeptide linker. To improve the thermostability of GCGR–G_s complex, forty-five residues (H433-F477) were truncated and the affinity tag HPC4 was added at the receptor C terminus. These modifications did not alter receptor pharmacology[3]. A dominant-negative $G\alpha_s$ ($DNG\alpha_s$) with 8 mutations (S54N, G226A, E268A, N271K, K274D, R280K, T284D and I285T) was generated by site-directed mutagenesis to stabilize interactions with the βγ subunits[4]. The modified rat Gβ1 and bovine Gγ2 were both cloned into a pFastBac vector. Nanobody 35 (Nb35) with a C-terminal 6× His-tag was cloned into the expression vector (pET28a) and used to limit G protein dissociation by binding at $G\alpha_s$-Gβ interface[5].

The human GIPR DNA (Genewiz) with one mutation ($T345^{6.44b}F$, class B1 GPCR numbering in superscript[6]) was cloned into pFastBac vector. The native signal peptide of GIPR was replaced by the HA signal peptide to enhance the receptor expression level. A BRIL fusion protein was added to the N terminus of the ECD with a TEV protease site and a 2 GSA linker between them[7]. Since it has been demonstrated that the whole C terminus is not necessary for GIPR−G protein complex interaction[8], forty-five amino acids (Q422-C466) were truncated at the C terminus. Besides, LgBiT[9] was attached to the end of helix 8 (H8) with a 15AA polypeptide linker between them, followed by a TEV protease cleavage site and an OMBP-

MBP tag. These modifications were inserted to facilitate the expression and purification of the receptor but not alter receptor pharmacological and functional properties[8]. The engineered G112, rat Gβ1-HiBiT, and bovine Gγ2 were cloned into the pFastBac vector, respectively.

Complex expression and purification

The Bac-to-Bac Baculovirus Expression System (Invitrogen) was used to generate high titer recombinant baculovirus for GLP-1R, GIPR, GCGR, $G\alpha_s$, Gβ1 and Gγ2. P0 viral stock was produced by transfecting 5 μg recombinant bacmids into *Sf*9 cells (2.5 mL, density of 1.5×10^6 cells/mL) for 96 h incubation and then used to produce P1 and P2 baculoviruses. Cell culture was grown in ESF 921 serum-free medium (Expression Systems) to a density of 3×10^6 cells/mL and then infected with four separate baculoviruses. The culture was collected by centrifugation 48 h after infection and cell pellets were stored at -80°C.

All purification steps were performed under ice-bath condition. For GLP-1R, the complex formation and purification were performed as described previously[10]. Briefly, the GLP-1R-LgBiT-2MBP, G112, Gβ1-HiBiT and Gγ2 were co-expressed by infecting *Sf*9 cells at a ratio of 1:1:1:1. Cell pellets were lysed in a buffer containing 20 mM HEPES (pH 7.4), 50 mM NaCl, 2 mM $MgCl_2$ supplemented with EDTA-free protease inhibitor cocktail (Bioteck). The complexes were formed by adding 10 μM retatrutide (GL Biochem), 10 μg/mL Nb35 (preparation method shown as below) and 25 mU/mL apyrase (New England Biolabs), incubating for 1 h at room temperature (RT). The complexes were then solubilized from membrane using 0.5% (w/v) lauryl maltose neopentyl glycol (LMNG, Anatrace) and 0.03% (w/v) cholesteryl hemisuccinate (CHS, Anatrace) for 2 h at 4°C. Supernatant was collected by ultracentrifugation at 65,000 *g* for 30 min at 4°C, and taken to bind with amylose resin for 2 h at 4°C. After packing, the resin was washed with 20 column volumes (CVs) of buffer containing 20 mM HEPES (pH 7.4), 100 mM NaCl, 10% (v/v) glycerol, 5 μM retatrutide, 25 μM Tris (2-carboxyethyl) phosphine hydrochloride (TCEP), 5 mM $MgCl_2$, 1 mM $MnCl_2$, 0.03% (w/v) LMNG, 0.01% (w/v) glyco-diosgenin (GDN) and 0.008% (w/v) CHS. TEV enzyme was added to the resin and kept at 4°C overnight to remove the 2MBP tag. The complex was eluted from the resin and concentrated to 500 μL using a 100 kDa MWCO Amicon Ultra Centrifugal Filter. Size-exclusion chromatography (SEC) was conducted by loading the protein sample to Superdex 200 Increase 10/300GL (GE Healthcare) to obtain the monomer complex. The

column was pre-equilibrated with 20 mM HEPES (pH 7.4), 100 mM NaCl, 5 μM retatrutide, 100 μM TCEP, 2 mM $MgCl_2$, 0.00075% (w/v) LMNG, 0.00025% (w/v) GDN, and 0.00015% (w/v) CHS.

For GIPR, recombinant baculovirus encoding the BRIL-GIPR(T345F)-LgBiT-2MBP, G112, Gβ1-HiBiT and Gγ2 were co-expressed in HighFive cells, at a ratio of 1:3:3:3 and purified as described previously[8]. For cell lysis, frozen cell pellets were thawed in a buffer containing 20 mM HEPES (pH 7.4), 100 mM NaCl and 10% glycerol supplemented with EDTA-free protease inhibitor cocktail (TargetMol). Cell membranes were then collected by ultracentrifugation at 4°C, 32,000 rpm for 30 min. Cell pellets were resuspended with a buffer consisting of 20 mM HEPES (pH 7.4), 100 mM NaCl, 10 mM $MgCl_2$, 1 mM $MnCl_2$, 100 μM TCEP, 25 mU/mL apyrase (Sigma-Aldrich), 10 μg/mL Nb35 and 10% glycerol. Fifteen μM retatrutide was added in the above system to assemble the retatrutide–GIPR–G_s complex at RT for 1.5 h. Then, the complex was solubilized with 0.5% (w/v) LMNG and 0.1% (w/v) CHS for 2 h at 4°C. The supernatant was then separated by ultracentrifugation at 32,000 rpm for 30 min and incubated with amylose resin for another 2 h at 4°C. The resin was washed with 5 CVs of washing buffer I containing 20 mM HEPES (pH 7.4), 100 mM NaCl, 5 mM $MgCl_2$, 1 mM $MnCl_2$, 25 μM TCEP, 0.1% (w/v) LMNG, 0.02% (w/v) CHS and 2 μM retatrutide, and then with 25 CVs of washing buffer II consisting of 20 mM HEPES (pH 7.4), 100 mM NaCl, 5 mM $MgCl_2$, 1 mM $MnCl_2$, 25 μM TCEP, 0.03% (w/v) LMNG, 0.01% (w/v) GDN, 0.008% (w/v) CHS and 2 μM retatrutide. TEV enzyme was added to the resin and kept at 4°C overnight to remove the 2MBP tag. The protein complex was eluted with 10 CVs of washing buffer II, and further purified by SEC using a Superose 6 Increase 10/300GL (Cytiva) equilibrated with a buffer containing 20 mM HEPES (pH 7.4), 100 mM NaCl, 2 mM $MgCl_2$, 0.00075% LMNG, 0.00025% GDN, 0.00002% CHS and 5 μM retatrutide. The peak fractions of the complex were collected and concentrated to approximately 8.3 mg/mL with a 100-kDa filter for cryo-EM analysis.

For GCGR, the GCGR-HPC4, DNGα_s, 6× His-tagged Gβ1 and Gγ2 were co-expressed at a ratio of 4:1:1:1 by infecting HighFive cells and purified as described previously[3, 11]. Cell pellets were resuspended in 20 mM HEPES (pH 7.4), 50 mM NaCl and 2 mM $MgCl_2$ with EDTA-free protease inhibitor cocktail. Following addition of 10 μM retatrutide, 10 μg/mL

Nb35 and 25 mU/mL apyrase, the suspension was incubated at RT for 1 h to promote the formation of complexes. Membranes were collected by ultracentrifugation (30,000 rpm) at 4 °C for 30 min, and solubilized in 0.5% (w/v) LMNG, 0.1% (w/v) CHS, 10 μM retatrutide, 2 mM $MgCl_2$ and 25 mU/mL apyrase for 3 h at 4°C. The supernatant was collected by ultracentrifugation at 30,000 rpm for 30 min and incubated with anti-HPC4 affinity resin in the presence of 2 mM $CaCl_2$ overnight at 4°C. The HPC4 resin was washed with 20 CVs of 20 mM HEPES (pH 7.4), 100 mM NaCl, 2 mM $MgCl_2$, 2 mM $CaCl_2$, 5 μM retatrutide, 0.02% (w/v) LMNG and 0.004% (w/v) CHS, and then eluted with 5 CVs of the above buffer by adding 6 mM EDTA and 10 μM retatrutide. The complexes were concentrated by a 100-kDa molecular weight cut-off concentrator and separated by SEC using a Superdex 200 Increase 10/300 column (GE Healthcare) with buffer containing 20 mM HEPES (pH 7.4), 100 mM NaCl, 2 mM $MgCl_2$, 0.01% (w/v) LMNG, 0.002% (w/v) CHS and 5 μM retatrutide. The complex samples were concentrated to 10 mg/mL for cryo-EM analysis.

Nb35 expression and purification

Nb35 was purified as described previously[1]. Briefly, Nb35 with a periplasmic signal peptide was expressed in *Escherichia coli* strain BL21 (DE3) and purified by nickel affinity chromatography. BL21 (DE3) cells were cultured in TB medium supplemented with 100 μg/mL ampicillin, 0.1% (w/v) glucose and 2 mM $MgCl_2$ at 37°C until OD600 reaching 0.8-1.2. The expression of Nb35 was induced by adding 1 mM IPTG and cultured at 28°C overnight and then cells were harvested by centrifugation at 4,000 rpm for 20 min. Nb35 was purified using a HiLoad 16/600 Superdex 75 column (GE Healthcare) with running buffer containing 20 mM HEPES and 100 mM NaCl, pH 7.4. The purified Nb35 was flash-frozen in 10% (vol/vol) glycerol by liquid nitrogen and stored at -80°C until use.

Cryo-EM data acquisition

The cryo-EM sample was prepared by plunge vitrification in liquid ethane on a Vitrobot Mark IV (ThermoFisher Scientific) with blotting chamber set to 4°C and 100% humidity. For the retatrutide-bound GLP-1R–G_s–Nb35 complexes, 3.5 μL sample solution was applied to glow-discharged holey carbon grids (Quantifoil, R1.2/1.3, Au 300 mesh) and blotted for 3 s before plunging. For the retatrutide-bound GIPR–G_s–Nb35 complexes, 2.5 μL sample solution was applied to a glow-discharged holey carbon grid (Quantifoil R1.2/1.3, Au 300 mesh) and blotted

for 3.5 s before plunging. For the retatrutide-bound GCGR–G_s–Nb35 complexes, 3.0 μL sample solution was applied to glow-discharged holey carbon grids (Quantifoil, R1.2/1.3, Au 300 mesh) and blotted for 3.5 s before plunging. Data were collected on a Titan Krios (ThermoFisher Scientific) 300 kV electron microscope equipped with a Gatan K3 Summit direct electron detector and serial EM3.7 was used to acquire cryo-EM images. The microscope was operated at a nominal magnification of 46,685× in counting mode, corresponding to a pixel size of 1.071 Å. For the GLP-1R and GCGR complexes, the total exposure time was set to 7.2 s with intermediate frames recorded every 0.2 s, resulting in an accumulated dose of 80 electrons per $Å^2$ fractionated into a movie stack of 36 frames with defocus range of -1.2 to -2.2 μm. For the GIPR complex, the total exposure time was set to 3.6 s, resulting in an accumulated dose of 80 electrons per $Å^2$ was fractionated into a movie stack of 36 frames with defocus range of -1.0 to -2.0 μm.

Cryo-EM data processing

For the retatrutide−GLP-1R−G_s complex, motion correction and CTF estimation for micrographs were done by patch motion correction and patch CTF estimation, respectively. Micrographs under 5 Å CTF resolution were removed, and 6,862,363 particles were auto picked by template picker referenced from a previously published map of GLP-1R−G_s complex (EMDB code: EMD-30867). After 2D classifications, particles from better classes were selected and classified by Hetero Refinement into 6 classes using *Ab-initio* volume generated from 200,000 particles as reference models. Then, 2,823,138 particles were selected for further 3D classification where soft masks around the receptor and ECD were used in focused classifications to improve the density in these regions. After two rounds of Hetero Refinement, non-uniform (NU) refinement and local refinement, a 2.68 Å map was obtained using 824,885 particles.

For the retatrutide−GIPR−G_s complex, 11,390 movies were recorded and processed using cryoSPARC v.4.4.121 with patch motion correction to restore the correct information of the original cryo-EM images. The contrast transfer function parameters were estimated using patch CTF estimation. A total of 8,199,888 particles were auto-picked by template picker referenced from a previously published map of GIPR−G_s complex (EMDB code: EMD-30860). After two rounds of 2D classification, three rounds of 3D classification, Hetero Refinement, NU-

refinement and local refinement, 413,303 particles were used to generate a map with an indicated global resolution of 3.26 Å.

For the retatrutide−GCGR−G_s complex, motion correction and CTF estimation for 6,446 micrographs were done by patch motion correction and patch CTF estimation, respectively. A total of 5,891,536 particles were auto picked by blob picker referenced from a previously published map of GCGR−G_s complex (EMDB code: EMD-0917). After 2D classifications, particles from better classes were selected and classified by Hetero Refinement into several classes using *Ab-initio* volume generated from 409,391 particles as reference model. Then, 1,308,476 particles were reselected for further 3D classification where soft masks around the receptor and ECD were used in focused classifications to improve the density in these regions. These particles after 4 rounds of Hetero Refinement, 3D reconstruction was performed by NU-refinement, and a 2.84 Å map was obtained using 554,835 particles.

Model building and refinement

The models of the retatrutide–GLP-1R–G_s, retatrutide−GIPR−G_s and retatrutide−GCGR−G_s complexes were built based on the cryo-EM structures of the GLP-1–GLP-1R–G_s (PDB ID: 6X18)[12], non-acylated tirzepatide–GIPR–G_s complex (PDB ID: 7VAB)[1] and GCG–GCGR–G_s (PDB ID: 6LMK)[11] complexes, respectively. The initial models were docked into the EM density map using UCSF Chimera v1.15[13] or UCSF Chimera X 1.6.1[13, 14], followed by iterative manual adjustment and rebuilding in COOT v0.9.2[15]. Real-space refinement was performed using Phenix v1.20[16]. The final refinement statistics were validated using the module comprehensive validation (cryo-EM) in Phenix v1.20[16]. Structural figures were prepared with UCSF Chimera v1.15, UCSF ChimeraX v1.0 and PyMOL v.2.1 (https://pymol.org/2/). The final refinement statistics are provided in **Supplementary Table S3**. Separate cryo-EM dada set was collected for retatrutide–GLP-1R–G_s (EMDB code: EMD-39631), which was referenced during the model building.

cAMP accumulation assay

HEK-293T cells were maintained at 37°C in 5% CO_2 incubator and seeded onto 6-well cell culture plates before transfection. After overnight culture, the cells were transiently transfected with various constructs using Lipofectamine 2000 transfection reagent (Invitrogen). After 24 h, the transfected cells were seeded onto 384-microtiter plates at a density of 3,000 cells per well

in stimulation buffer (HBSS supplemented with 5 mM HEPES, 0.1% (w/v) casein and 0.5 mM 3-isobutyl-1-methylxanthine). Different concentrations of retatrutide in stimulation buffer were added, and the stimulation lasted for 40 min. The reaction was stopped by addition of cAMP detection buffer containing Eu-cAMP tracer and ULight-anti-cAMP. Plates were then incubated for 60 min at RT. Time-resolved FRET signals were measured at 620 nm and 665 nm, respectively, by an EnVision multilabel plate reader (PerkinElmer). Data were analyzed in GraphPad PRISM 9.5 and all values were normalized to that of the wild-type (WT).

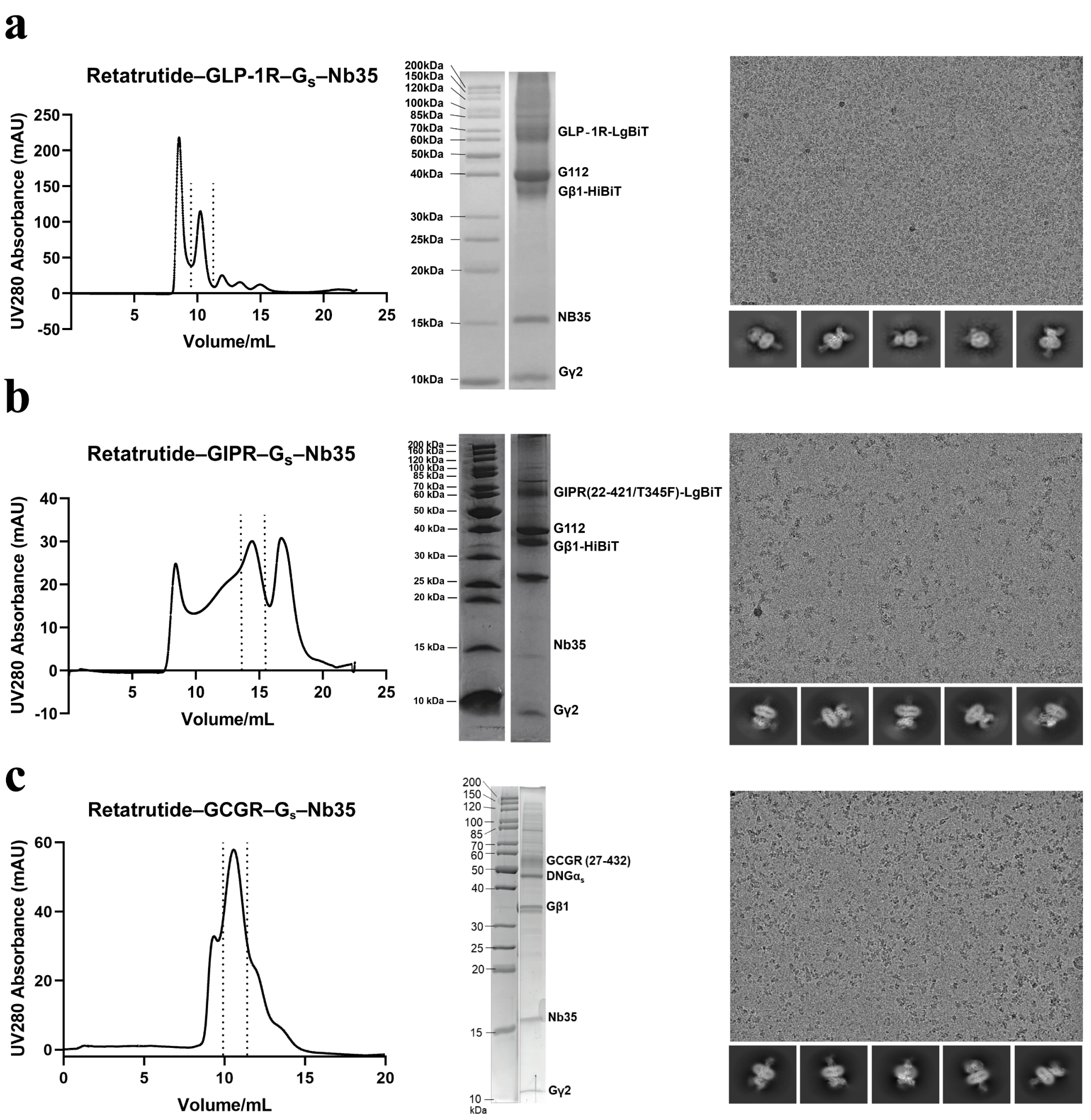

Supplementary Fig. 1 Purification and cryo-EM imaging of the retatrutide–GLP-1R–G_s (a), retatrutide–GIPR–G_s (b) and retatrutide–GCGR–G_s (c) complexes.

a Left, analytical size-exclusion chromatography (SEC) and SDS-PAGE/Coomassie blue of the retatrutide–GLP-1R–G_s complex, fractions of the complex are shown by dashed lines; Right, representative cryo-EM micrograph and two-dimensional (2D) class averages of the selected particles. **b** Left, SEC and SDS-PAGE/Coomassie blue of the retatrutide–GIPR–G_s complex, fractions of the complex are shown by dashed lines; Right, representative cryo-EM micrograph and 2D class averages of the selected particles. **c** Left, SEC and SDS-PAGE/Coomassie blue of the retatrutide–GCGR–G_s complex, fractions of the complex are shown by dashed lines; Right, representative cryo-EM micrograph and 2D class averages of the selected particles.

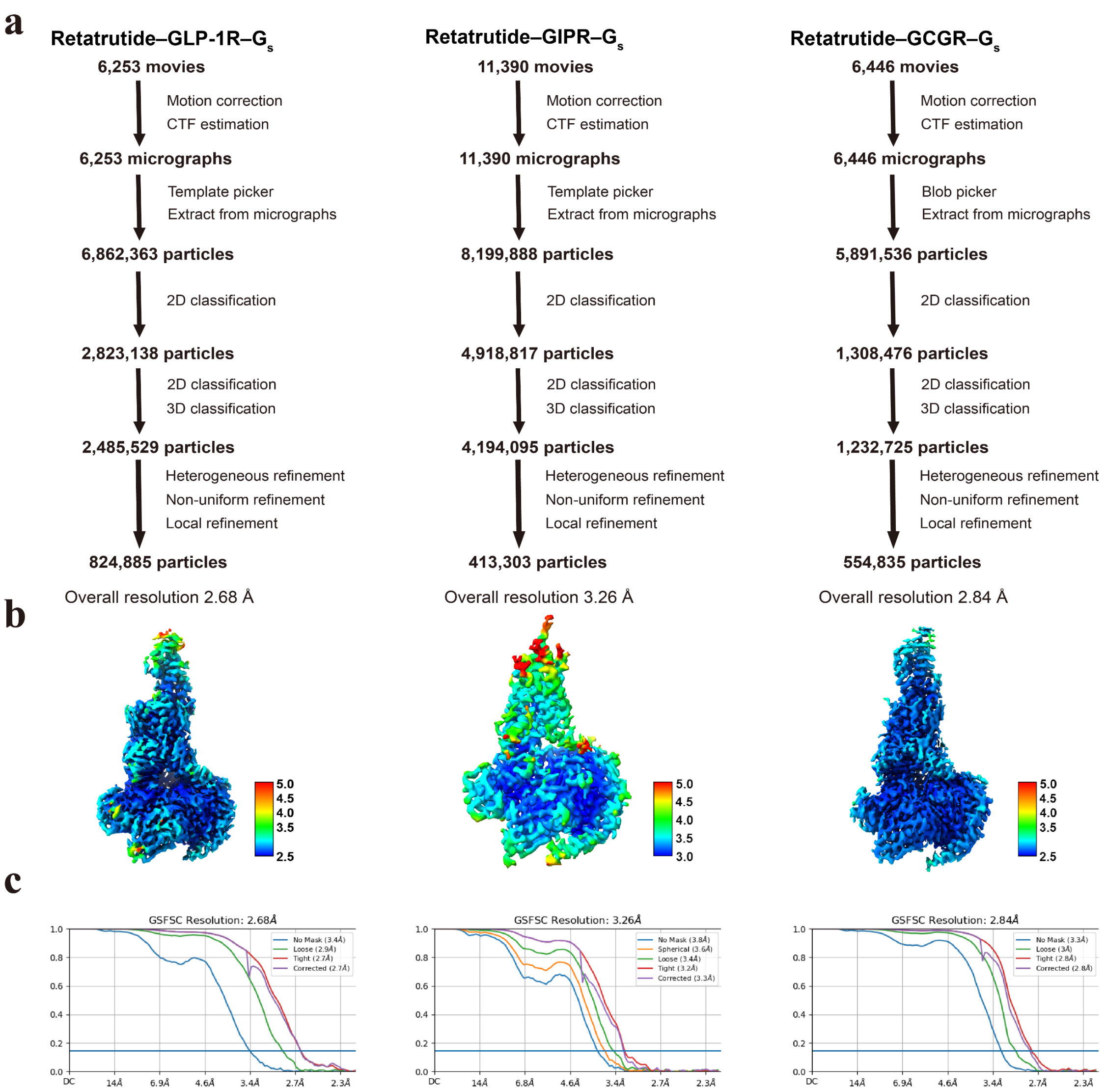

Supplementary Fig. 2 Cryo-EM data processing and validation of the retatrutide–GLP-1R–G_s, retatrutide–GIPR–G_s and retatrutide–GCGR–G_s complexes.

a Cryo-EM data processing flow charts for the retatrutide–GLP-1R–G_s (left), retatrutide–GIPR–G_s (middle) and retatrutide–GCGR–G_s (right) complexes. **b** Density maps colored by local resolution for the retatrutide–GLP-1R–G_s (left), retatrutide–GIPR–G_s (middle) and retatrutide–GCGR–G_s (right) complexes. **c** Gold standard Fourier shell correlation (FSC) curves of overall refined structures, indicating the global resolution at 0.143 FSC threshold.

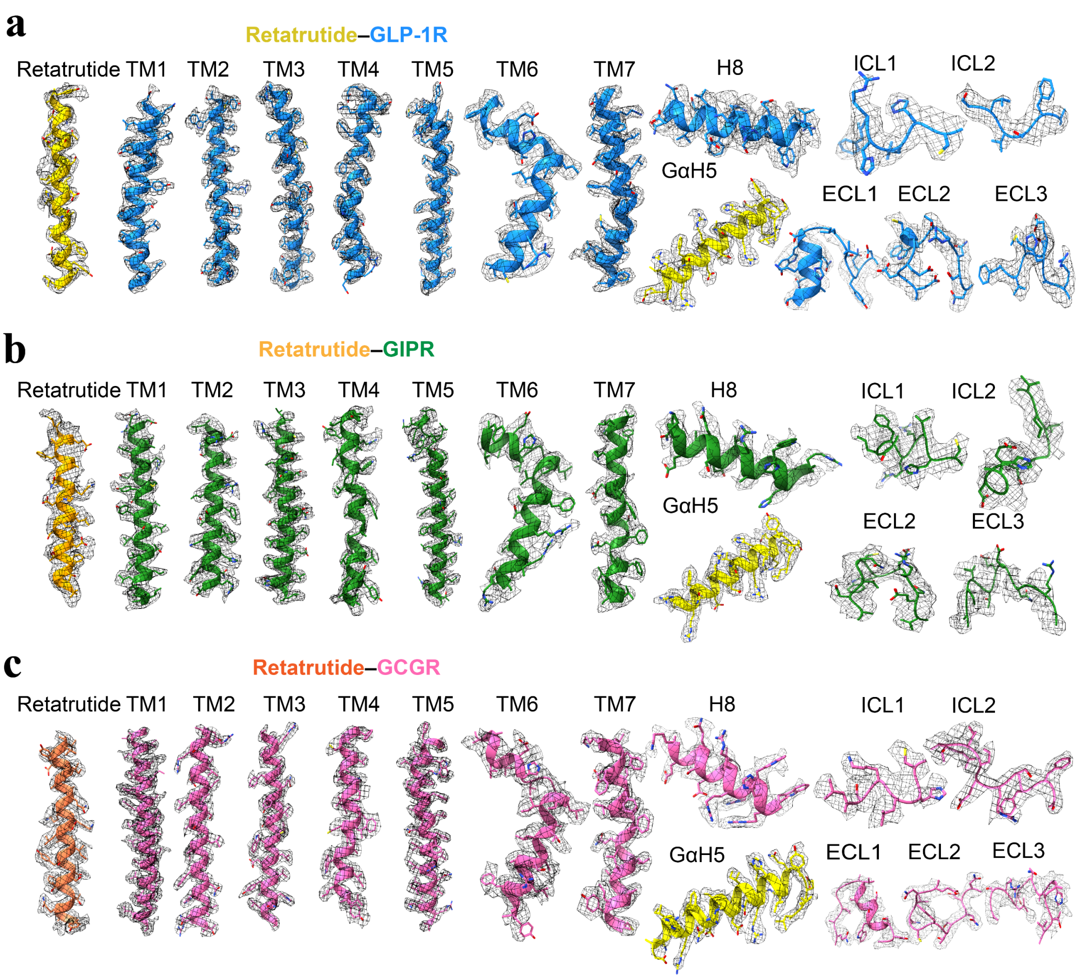

Supplementary Fig. 3 Near-atomic resolution model of the three complexes in the cryo-EM density maps.

a EM density map and model of the retatrutide–GLP-1R–G_s complex are shown for all seven-transmembrane α-helices (TMs 1-7), helix 8 (H8), intracellular loops 1 and 2 (ICL1 and ICL2), all extracellular loops (ECL1-ECL3), retatrutide and the α5-helix of the $G\alpha_s$ (GαH5). **b** EM density map and model of the retatrutide–GIPR–G_s complex are shown for TMs 1-7, H8, ICL1, ICL2, ECL2, ECL3, retatrutide and GαH5. **c** EM density map and model of the retatrutide–GCGR–G_s complex are shown for TMs 1-7, H8, ICL1, ICL2, ECL1-ECL3, retatrutide and GαH5.

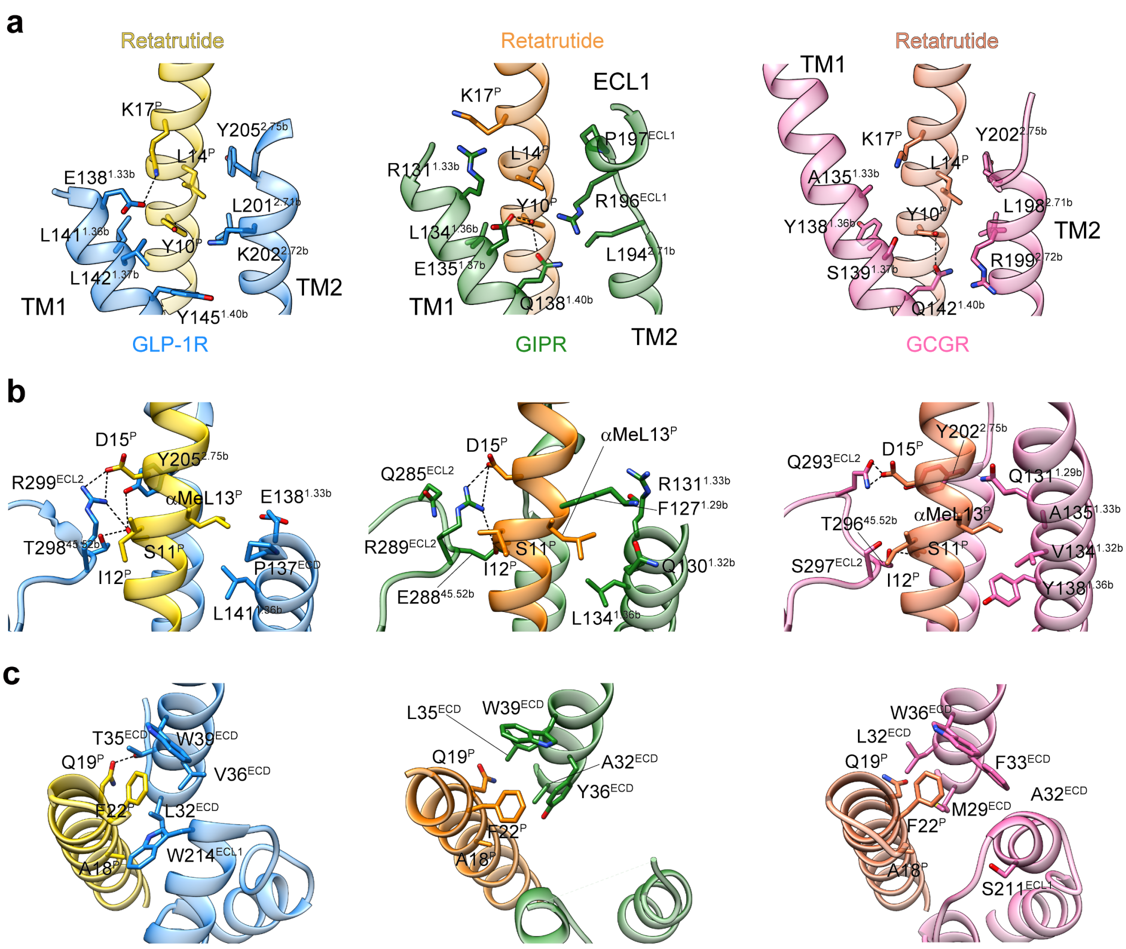

Supplementary Fig. 4 Retatrutide-binding mode comparison among GLP-1R, GIPR and GCGR.

a Interactions between three residues (Y10^{P}, L14^{P} and K17^{P}) at the middle region of retatrutide and the TMD of GLP-1R (left), GIPR (middle) or GCGR (right). **b** Interactions between the three residues (S11^{P}, αMeL13^{P} and D15^{P}) at the middle region of retatrutide and the TM1/TM2 and ECL2 of GLP-1R (left), GIPR (middle) or GCGR (right). **c** Interactions between the three residues (A18^{P}, Q19^{P} and F22^{P}) at the C-terminal region of retatrutide and the ECD and ECL1 of GLP-1R (left), GIPR (middle) or GCGR (right). Residues involved in interactions are shown as sticks. The polar contacts are shown as black dashed lines.

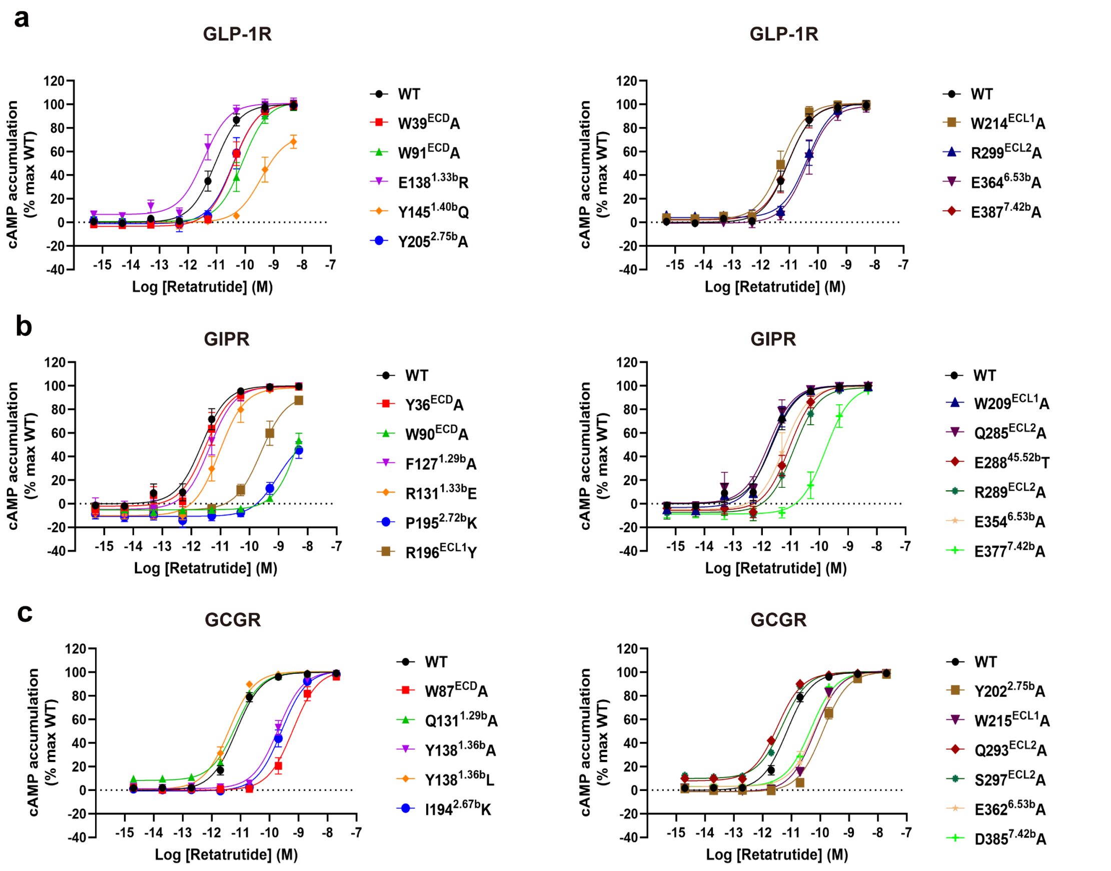

Supplementary Fig. 5 Effects of receptor mutation on retatrutide-induced cAMP accumulation. a-c, Signaling profiles of GLP-1R (**a**), GIPR (**b**) and GCGR (**c**) mutants. cAMP accumulation was measured in wild-type (WT) and single-point mutated GLP-1R, GIPR or GCGR expressing HEK-293T cells. cAMP levels were normalized to the maximum response of the WT and dose-response curves were analyzed using a three-parameter logistic equation. Data were generated and graphed as means ± S.E.M. of at least three independent experiments performed in quadruplicate.

Supplementary Table S1 Completed clinical trials of retatrutide.

No.	Study type	Authors/ Sponsor	NCT detail	Clinical effect	Adverse event
1	Phase 1a: single-ascending dose clinical trial	Coskun T *et al*[17] (PMID: 35985340)	NCT03841630 45 participants 4 months (03/13/2019–07/25/2019)	Retatrutide dose-dependently reduced C57/Bl6 diet-induced obese (DIO) mice body weight (ED_{50}: 4.73 nmol/kg) and calorie intake. For humans, decreases in body weight were greatest at the highest doses of 4.5 mg (up to –2.93 kg) and 6 mg (up to –3.52 kg).	Most treatment-related adverse events were mild, such as vomiting and nausea reported by 2 participants in the 4.5 mg retatrutide dose group.
2	Phase 1b: multiple-ascending dose trial	Urva S *et al*[18] (PMID: 36354040)	NCT04143802 72 participants 12 months (12/18/2019–12/28/2020)	Retatrutide showed glycemic control efficacy and clinically decreases in fasting and postprandial plasma glucose as well as HbA1c compared to placebo. Body weight reduction was greater in the three highest dose groups (–4.71 kg for the 3 mg group, –7.83 kg for the 3/6 mg group, and –8.96 kg for the 3/6/9/12 mg group).	The 3, 6, 9 and 12 mg groups reported gastrointestinal-related adverse events compared to the 1.5 mg dose group.
3	Phase 2 obesity trial	Jastreboff AM *et al*[19] (PMID: 37888927)	NCT04881760 338 participants 18 months (05/20/2021–11/22/2022)	The least-squares mean percentage change in body weight at 24 weeks in the retatrutide groups was –12.9% in the combined 4-mg group, –17.3% in the combined 8-mg group, and –17.5% in the 12-mg group, compared to –1.6% in the placebo group. At 48 weeks, the least-squares mean percentage change in the retatrutide groups was –17.1% in the combined 4-mg group, –22.8% in the combined 8-mg group, and –24.2% in the 12-mg group, compared to –2.1% in the placebo group. All the participants treated at a dose of 8 mg or 12 mg had a weight reduction of 5% or more. With the 12 mg dose, 9 of 10 participants lost 10% or more of their baseline weight, nearly two thirds lost 20% or more, nearly half lost 25% or more, and a quarter lost 30% or more.	The most common adverse events were gastrointestinal and were mild to moderate in severity, partially mitigated with a lower starting dose (2 mg *vs*. 4 mg). Dose-dependent increases in heart rate peaked at 24 weeks and declined thereafter.
4	Phase 2: type 2 diabetes trial	Rosenstock J *et al*[20] (PMID: 37385280)	NCT04867785 281 participants 19 months (03/13/2021–10/27/2022)	Retatrutide showed clinically improvements in glycemic control, dose-dependent reduction of blood pressure, triglycerides (~35%) and non-HDL cholesterol. Up to 63% of retatrutide-treated participants lost at least 15% of body weight at 36 weeks.	Gastrointestinal events were the most frequently reported adverse events in 35% participants and occurred more frequently with the 4 mg starting dose group.

Supplementary Table S2 Ongoing clinical trials of retatrutide.

No.	Study type	Sponsor	NCT detail	Current status
1	Phase 3: obesity and established cardiovascular disease (CVD) trial	Eli Lilly	NCT05882045<br>1,800 participants<br>113 weeks	A study of retatrutide (LY3437943) in participants with obesity and CVD (TRIUMPH-3)<br>Recruitment status: recruiting<br>First posted: May 31, 2023<br>Actual study starting date: May 30, 2023<br>Estimated primary completion date: January 20, 2026<br>Estimated study completion date: February 17, 2026<br>URL: https://classic.clinicaltrials.gov/ct2/show/NCT05882045
2	Phase 3: obesity or overweight without type 2 diabetes mellitus (T2DM) trial	Eli Lilly	NCT05929066<br>2,100 participants<br>89 weeks	A study of retatrutide (LY3437943) in participants who have obesity or overweight (TRIUMPH-1)<br>Recruitment status: recruiting<br>First posted: July 3, 2023<br>Actual study starting date: July 10, 2023<br>Estimated primary completion date: April 15, 2026<br>Estimated study completion date: May 13, 2026<br>URL: https://classic.clinicaltrials.gov/ct2/show/NCT05929066
3	Phase 3: obesity or overweight with obstructive sleep apnea (OSA) trial	Eli Lilly	NCT05929079<br>1,000 participants<br>89 weeks	A study of retatrutide (LY3437943) in participants who have obesity or overweight with OSA (J1I-MC-GSA2)<br>Recruitment status: recruiting<br>First posted: July 3, 2023<br>Actual study starting date: July 11, 2023<br>Estimated primary completion date: May 3, 2026<br>Estimated study completion date: May 31, 2026<br>URL: https://classic.clinicaltrials.gov/ct2/show/ NCT05929079
4	Phase 3: obesity or overweight with osteoarthritis (OA) of the knee trial	Eli Lilly	NCT05931367<br>405 participants<br>77 weeks	A study of retatrutide (LY3437943) in participants who have obesity or are overweight with OA of the knee.<br>Recruitment status: recruiting<br>First posted: July 5, 2023<br>Actual study starting date: August 1, 2023<br>Estimated primary completion date: February 6, 2026<br>Estimated study completion date: March 8, 2026<br>URL: https://classic.clinicaltrials.gov/ct2/show/ NCT05931367
5	Phase 3: overweight or obesity and chronic kidney disease (CKD), with or without T2DM trial	Eli Lilly	NCT05936151<br>120 participants<br>31 weeks	A study of retatrutide (LY3437943) in participants who have overweight or obesity and CKD with or without T2DM.<br>Recruitment status: recruiting<br>First posted: July 7, 2023<br>Actual study starting date: July 20, 2023<br>Estimated primary completion date: November 25, 2025<br>Estimated study completion date: November 25, 2025<br>URL: https://classic.clinicaltrials.gov/ct2/show/ NCT05936151

6	Phase 3: T2DM with multi-drug combination trial	Eli Lilly	NCT06260722<br>1,250 participants<br>26 months	A study of retatrutide (LY3437943) in participants who have T2DM and inadequate glycemic control with metformin with or without SGLT2 inhibitor (TRANSCEND-T2D-2).<br>Recruitment status: recruiting<br>First posted: February 15, 2024<br>Actual study starting date: February 21, 2024<br>Estimated primary completion date: December 11, 2026<br>Estimated study completion date: March 30, 2027<br>URL: https://classic.clinicaltrials.gov/ct2/show/ NCT06260722
7	Phase 3: T2DM and moderate or severe renal impairment with multi-drug combination trial	Eli Lilly	NCT06297603<br>320 participants<br>14 months	A study of retatrutide (LY3437943) in participants who have T2DM and moderate or severe renal impairment with inadequate glycemic control on basal insulin with or without metformin and/or SGLT2 inhibitor.<br>Recruitment status: recruiting<br>First posted: March 7, 2024<br>Actual study starting date: March 15, 2024<br>Estimated primary completion date: September 11, 2026<br>Estimated study completion date: October 9, 2026<br>URL: https://classic.clinicaltrials.gov/ct2/show/ NCT06297603
8	Phase 3: Obesity and atherosclerotic cardiovascular disease with or without CKD trial	Eli Lilly	NCT06383390<br>10,000 participants<br>5 years	A study of retatrutide (LY3437943) in participants who have obesity and atherosclerotic cardiovascular disease with or without CKD.<br>Recruitment status: recruiting<br>First posted: April 25, 2024<br>Actual study starting date: April 30, 2024<br>Estimated primary completion date: February 2029<br>Estimated study completion date: February 2029
9	Phase 3: T2DM with inadequate glycemic control trial	Eli Lilly	NCT06354660<br>480 participants<br>11 months	A study of retatrutide (LY3437943) in participants who have T2DM and inadequate glycemic control with diet and exercise alone (TRANSCEND-T2D-1).<br>Recruitment status: recruiting<br>First posted: April 9, 2024<br>Actual study starting date: April 10, 2024<br>Estimated primary completion date: June 2026<br>Estimated study completion date: July 2026<br>URL: https://classic.clinicaltrials.gov/ct2/show/ NCT06354660

CKD, chronic kidney disease; CVD, cardiovascular disease; OA, osteoarthritis; OSA, obstructive sleep apnea; T2DM, type 2 diabetes mellitus.

Supplementary Table S3 Cryo-EM data collection, refinement and validation statistics.

	Retatrutide–GLP-1R–G_s–Nb35 complex	Retatrutide–GIPR–G_s–Nb35 complex	Retatrutide–GCGR–G_s–Nb35 complex
**Data collection and processing**			
Magnification	46,685	46,685	46,685
Voltage (kV)	300	300	300
Electron exposure (e^-/Å^2)	80	80	80
Defocus range (μm)	-1.2 to -2.2	-1.0 to -2.0	-1.2 to -2.2
Pixel size (Å)	1.071	1.071	1.071
Symmetry imposed	C1	C1	C1
Final particle images (no.)	824,885	413,303	554,835
Map resolution (Å)	2.68	3.26	2.84
FSC threshold	0.143	0.143	0.143
**Refinement**			
Initial model used (PDB ID)	6X18	7VAB	6LMK
Model resolution (Å)	2.10	3.20	3.70
FSC threshold	0.5	0.5	0.5
Model resolution range (Å)	2.5–6.0	2.5–6.0	2.5–6.0
Model composition			
Non-hydrogen atoms	9,231	8,619	8,937
Protein residues	1,160	1,082	1,157
B factors (Å^2)			
Protein	287.11	119.90	184.37
R.m.s. deviations			
Bond lengths (Å)	0.005	0.004	0.003
Bond angles (°)	1.291	0.576	0.512
Validation			
MolProbity score	2.47	1.80	2.00
Clash score	11.34	7.88	4.62
Poor rotamers (%)	7.93	0.33	3.79
Ramachandran plot			
Favored (%)	96.46	94.69	95.39
Allowed (%)	3.36	5.12	4.61
Disallowed (%)	0.00	0.19	0.00

Supplementary Table S4 Interaction between retatrutide and GLP-1R, GIPR or GCGR.

Retatrutide	GLP-1R	GIPR	GCGR
Y1^{P}	Hydrogen bond with Q234$^{3.37b}$<br>Salt bridge with E364$^{6.53b}$ and E387$^{7.42b}$<br>Stacking with W306$^{5.36b}$<br>Hydrophobic contacts with V237$^{3.40b}$	Hydrogen bond with Q224$^{3.37b}$<br>Salt bridge with E354$^{6.53b}$ and E377$^{7.42b}$<br>Stacking with W296$^{5.36b}$<br>Hydrophobic contacts with V227$^{3.40b}$	Salt bridge with E362$^{6.53b}$ and D385$^{7.42b}$<br>Stacking with W304$^{5.36b}$<br>Hydrophobic contacts with I235$^{3.40b}$ and V311$^{5.43b}$
Aib2^{P}	Hydrophobic contacts with L384$^{7.39b}$, E387$^{7.42b}$ and L388$^{7.43b}$	Hydrophobic contacts with L374$^{7.39b}$, E377$^{7.42b}$ and I378$^{7.43b}$	Hydrophobic contacts with L382$^{7.39b}$, D385$^{7.42b}$ and L386$^{7.43b}$
Q3^{P}	Hydrogen bond with Y148$^{1.43b}$ and Y152$^{1.47b}$	Hydrogen bond with Y141$^{1.43b}$ and Y145$^{1.47b}$	Hydrogen bond with Y145$^{1.43b}$ and Y149$^{1.47b}$
G4^{P}	Hydrophobic contacts with M233$^{3.36b}$ and W306$^{5.36b}$	Hydrophobic contacts with W296$^{5.36b}$	Hydrophobic contacts with M231$^{3.36b}$ and W304$^{5.36b}$
T5^{P}	Hydrogen bond with D372^{ECL3}<br>Hydrophobic contacts with L384$^{7.39b}$	Hydrophobic contacts with L374$^{7.39b}$	Hydrogen bond with D370$^{6.61b}$
F6^{P}	Stacking with Y148$^{1.43b}$<br>Hydrophobic contacts with L141$^{1.36b}$, L144$^{1.39b}$ and L388$^{7.43b}$	Stacking with Y141$^{1.43b}$<br>Hydrophobic contacts with L134$^{1.36b}$, L137$^{1.39b}$ and L378$^{7.43b}$	Stacking with Y138$^{1.36b}$ and Y145$^{1.43b}$<br>Hydrophobic contacts with L386$^{7.43b}$
T7^{P}	Hydrogen bond with K197$^{2.67b}$<br>Hydrophobic contacts with F230$^{3.33b}$, M233$^{3.36b}$ and T298$^{45.52b}$	Hydrogen bond with E288$^{45.52b}$ and R190$^{2.67b}$	Hydrophobic contacts with M231$^{3.36b}$ and T296$^{45.52b}$
S8^{P}	Hydrogen bond with N300^{ECL2}	Hydrogen bond with N290^{ECL2}	Hydrogen bond with N298^{ECL2}
D9^{P}	Salt bridge with R380$^{7.35b}$	Salt bridge with R370$^{7.35b}$	Hydrogen bond with Q374^{ECL3}
Y10^{P}	Hydrophobic contacts with L141$^{1.36b}$	Hydrogen bond with E135$^{1.37b}$ and Q138$^{1.40b}$<br>Hydrophobic contacts with L134$^{1.36b}$	Hydrogen bond with Q142$^{1.40b}$<br>Hydrophobic contacts with Y138$^{1.36b}$
S11^{P}	Hydrogen bond with Y205$^{2.75b}$, T298$^{45.52b}$ and R299^{ECL2}	Hydrogen bond with E288$^{45.52b}$ and R289^{ECL2}	Hydrogen bond with T296$^{45.52b}$
I12^{P}	Hydrophobic contacts with R299^{ECL2}	Hydrophobic contacts with R289^{ECL2}	
αMeL13^{P}	Hydrophobic contacts with L141$^{1.36b}$	Hydrophobic contacts with R131$^{1.33b}$ and	Hydrophobic contacts with A135$^{1.33b}$ and Y138$^{1.36b}$

		L134$^{1.36b}$	
L14^{P}	Hydrophobic contacts with L201$^{2.71b}$ and Y205$^{2.75b}$	Hydrophobic contacts with R131$^{1.33b}$ and R196^{ECL1}	Hydrophobic contacts with L198$^{2.71b}$ and Y202$^{2.75b}$
D15^{P}	Salt bridge with R299^{ECL2}<br>Hydrogen bond with L32^{ECD}	Salt bridge with R289^{ECL2}<br>Hydrogen bond with A32^{ECD}	Hydrogen bond with M29^{ECD} and Q293^{ECL2}
K16^{P}		Hydrophobic contacts with F127$^{1.29b}$	
K17^{P}	Salt bridge with E138$^{1.33b}$		
A18^{P}	Hydrophobic contacts with L32^{ECD} and W214^{ECL1}	Hydrophobic contacts with P197^{ECL1}	Hydrophobic contacts with M29^{ECD}
Q19^{P}	Hydrogen bond with T35^{ECD}<br>Hydrophobic contacts with L32^{ECD}	Hydrophobic contacts with A32^{ECD} and L35^{ECD}	Hydrophobic contacts with M29^{ECD} and L32^{ECD}
Aib20^{P}	Hydrophobic contacts with W91^{ECD}		Hydrophobic contacts with W87^{ECD} and M123^{ECD}
A21^{P}	Hydrophobic contacts with W214^{ECL1}		
F22^{P}	Stacking with W39^{ECD} and W214^{ECL1}<br>Hydrophobic contacts with V36^{ECD}	Stacking with Y36^{ECD} and W39^{ECD}	Stacking with F33^{ECD} and W36^{ECD}<br>Hydrophobic contacts with M29^{ECD} and L32^{ECD}
I23^{P}	Hydrophobic contacts with L89^{ECD} and P90^{ECD}		Hydrophobic contacts with L32^{ECD}, L85^{ECD} and W87^{ECD}
Y25^{P}	Stacking with W214^{ECL1}		Hydrogen bond with G207^{ECL1}<br>Hydrophobic contacts with I206^{ECL1}
L26^{P}	Hydrophobic contacts with W39^{ECD} and Y88^{ECD}		Hydrophobic contacts with W36^{ECD}, K64^{ECD} and Y84^{ECD}
L27^{P}	Hydrogen bond with R121^{ECD}<br>Hydrophobic contacts with Y69^{ECD} and L123^{ECD}		Hydrogen bond with R116^{ECD}<br>Hydrophobic contacts with Y65^{ECD}

Supplementary Table S5 Effects of the ligand-binding pocket residue mutation on retatrutide-induced cAMP signaling at GLP-1R, GIPR or GCGR.

Receptor	Mutation	pEC$_{50}$ ± S.E.M.	E$_{max}$ ± S.E.M. (% WT)
GLP-1R	WT	11.05 ± 0.08	100.00 ± 2.75
	W39^{ECD}A	10.41 ± 0.08**	101.39 ± 3.58
	W91^{ECD}A	10.08 ± 0.11****	102.36 ± 4.73
	E138$^{1.33b}$R	11.45 ± 0.11	100.84 ± 3.40
	Y145$^{1.40b}$Q	9.45 ± 0.15****	73.89 ± 5.95***
	Y205$^{2.75b}$A	10.41 ± 0.11**	101.35 ± 4.70
	W214^{ECL1}A	11.25 ± 0.10	100.98 ± 3.27
	R299^{ECL2}A	10.38 ± 0.10***	101.97 ± 3.92
	E364$^{6.53b}$A	10.39 ± 0.12***	98.84 ± 4.77
	E387$^{7.42b}$A	11.03 ± 0.10	100.40 ± 3.44
GIPR	WT	11.64 ± 0.10	100.00 ± 2.84
	Y36^{ECD}A	11.51 ± 0.12	99.10 ± 3.86
	W90^{ECD}A	N.D.	N.D.
	F127$^{1.29b}$A	11.38 ± 0.12	99.67 ± 4.00
	R131$^{1.33b}$E	11.03 ± 0.10**	98.44 ± 3.88
	P195$^{2.72b}$K	N.D.	N.D.
	R196^{ECL1}Y	9.61 ± 0.11****	92.16 ± 5.36
	W209^{ECL1}A	11.70 ± 0.11	100.17 ± 3.21
	Q285^{ECL2}A	11.76 ± 0.15	100.34 ± 4.02
	E288$^{45.52b}$T	11.05 ± 0.08**	100.81 ± 2.93
	R289^{ECL2}A	10.90 ± 0.11***	98.56 ± 4.01
	E354$^{6.53b}$A	11.23 ± 0.12	99.43 ± 4.28
	E377$^{7.42b}$A	9.78 ± 0.13****	99.83 ± 6.38
GCGR	WT	11.15 ± 0.05	100.00 ± 1.46
	W87^{ECD}A	9.20 ± 0.08****	101.97 ± 3.51
	Q131$^{1.29b}$A	11.17 ± 0.06	100.15 ± 1.51
	Y138$^{1.36b}$A	9.71 ± 0.05****	101.28 ± 2.08
	Y138$^{1.36b}$L	11.40 ± 0.05*	100.64 ± 1.39
	I194$^{2.67b}$K	9.58 ± 0.06****	101.55 ± 2.54
	Y202$^{2.75b}$A	9.91 ± 0.05****	100.47 ± 2.15
	K205^{ECL1}A	11.03 ± 0.07	100.00 ± 2.06
	W215^{ECL1}A	10.18 ± 0.06****	101.34 ± 2.03
	Q293^{ECL2}A	11.49 ± 0.04**	99.73 ± 0.94
	S297^{ECL2}A	11.31 ± 0.05	100.48 ± 1.34
	E362$^{6.53b}$A	10.18 ± 0.07****	100.39 ± 2.54
	D385$^{7.42b}$A	10.32 ± 0.04****	100.58 ± 1.33

cAMP levels were normalized to the maximum response of the wild-type (WT) and dose-response curves were analyzed using a three-parameter logistic equation to obtain pEC$_{50}$ and E$_{max}$ values. The experiments were carried out independently at least three times. Data shown are means ± S.E.M. One-way ANOVA was used to determine statistical difference. N.D., values that could not be determined due to incomplete curve fits. *P< 0.05, **P< 0.01, ***P< 0.001 and ****P<0.0001.

References

1 Zhao F, Zhou Q, Cong Z *et al.* Structural insights into multiplexed pharmacological actions of tirzepatide and peptide 20 at the GIP, GLP-1 or glucagon receptors. *Nat Commun* 2022; **13**:1057.

2 Zhou F, Ye C, Ma X *et al.* Molecular basis of ligand recognition and activation of human V2 vasopressin receptor. *Cell Res* 2021; **31**:929-931.

3 Chang R, Zhang X, Qiao A *et al.* Cryo-electron microscopy structure of the glucagon receptor with a dual-agonist peptide. *J Biol Chem* 2020; **295**:9313-9325.

4 Liang YL, Khoshouei M, Glukhova A *et al.* Phase-plate cryo-EM structure of a biased agonist-bound human GLP-1 receptor-Gs complex. *Nature* 2018; **555**:121-125.

5 Rasmussen SG, DeVree BT, Zou Y *et al.* Crystal structure of the beta2 adrenergic receptor-G_s protein complex. *Nature* 2011; **477**:549-555.

6 Wootten D, Simms J, Miller LJ, Christopoulos A, Sexton PM. Polar transmembrane interactions drive formation of ligand-specific and signal pathway-biased family B G protein-coupled receptor conformations. *Proc Natl Acad Sci U S A* 2013; **110**:5211-5216.

7 Liang YL, Zhao P, Draper-Joyce C *et al.* Dominant negative G proteins enhance formation and purification of agonist-GPCR-G protein complexes for structure determination. *ACS Pharmacol Transl Sci* 2018; **1**:12-20.

8 Zhao F, Zhang C, Zhou Q *et al.* Structural insights into hormone recognition by the human glucose-dependent insulinotropic polypeptide receptor. *Elife* 2021; **10**: e68719.

9 Dixon AS, Schwinn MK, Hall MP *et al.* NanoLuc complementation reporter optimized for accurate measurement of protein interactions in cells. *ACS Chem Biol* 2016; **11**:400-408.

10 Cong Z, Zhou Q, Li Y *et al.* Structural basis of peptidomimetic agonism revealed by small- molecule GLP-1R agonists Boc5 and WB4-24. *Proc Natl Acad Sci U S A* 2022; **119**:e2200155119.

11 Qiao A, Han S, Li X *et al.* Structural basis of G(s) and G(i) recognition by the human glucagon receptor. *Science* 2020; **367**:1346-1352.

12 Zhang X, Belousoff MJ, Zhao P *et al.* Differential GLP-1R binding and activation by peptide and non-peptide agonists. *Mol Cell* 2020; **80**:485-500 e487.

13 Pettersen EF, Goddard TD, Huang CC *et al.* UCSF Chimera--a visualization system for exploratory research and analysis. *J Comput Chem* 2004; **25**:1605-1612.

14 Meng EC, Goddard TD, Pettersen EF *et al.* UCSF ChimeraX: Tools for structure building and analysis. *Protein Sci* 2023; **32**:e4792.

15 Emsley P, Cowtan K. Coot: model-building tools for molecular graphics. *Acta Crystallogr D Biol Crystallogr* 2004; **60**:2126-2132.

16 Adams PD, Afonine PV, Bunkoczi G *et al.* PHENIX: a comprehensive Python-based system for macromolecular structure solution. *Acta Crystallogr D Biol Crystallogr* 2010; **66**:213-221.

17 Coskun T, Urva S, Roell WC *et al.* LY3437943, a novel triple glucagon, GIP, and GLP-1 receptor agonist for glycemic control and weight loss: From discovery to clinical proof of concept. *Cell Metabo* 2022; **34**:1234-1247.

18 Urva S, Coskun T, Loh MT *et al.* LY3437943, a novel triple GIP, GLP-1, and glucagon receptor agonist in people with type 2 diabetes: a phase 1b, multicentre, double-blind, placebo-controlled, randomised, multiple-ascending dose trial. *Lancet* 2022; **400**:1869-1881.

19 Bisson A, Fauchier G, Fauchier L. Triple-Hormone-Receptor Agonist Retatrutide for Obesity. *N Engl J Med* 2023; **389**:1628-1630.

20 Rosenstock J, Frias J, Jastreboff AM *et al.* Retatrutide, a GIP, GLP-1 and glucagon receptor agonist, for people with type 2 diabetes: a randomised, double-blind, placebo and active-controlled, parallel-group, phase 2 trial conducted in the USA. *Lancet* 2023; **402**:529-544.
